# Preceptorship contributions to the development of clinical and managerial skills in nursing residency

**DOI:** 10.1590/0034-7167-2022-0510

**Published:** 2023-05-12

**Authors:** Mayssa da Conceição Araújo, Marina Peduzzi, Nathália Romeu de Mazzi, Camila Mendes da Silva Souza, Valéria Marli Leonello

**Affiliations:** I Universidade de Brasília. Brasília, Distrito Federal, Brazil; II Universidade de São Paulo. São Paulo, São Paulo, Brazil

**Keywords:** Preceptorship, Inservice Training, Nursing, Professional Competence, Clinical Competence., Preceptoría, Capacitación en Servicio, Enfermería, Competencia Profesional, Competencia Clínica., Preceptoria, Capacitação em Serviço, Enfermagem, Competência Profissional, Competência Clínica.

## Abstract

**Objectives:**

to analyze the experience of resident nurses with preceptorship contributions to the development of common clinical and managerial skills acquired in pedagogical projects.

**Methods:**

exploratory qualitative research developed in two stages: document analysis of pedagogical projects and semi-structured interviews with residents. Content analysis was carried out based on the framework of the nurse’s work process and skills.

**Results:**

the pedagogical projects of the three programs foresee the development of common skills, mostly clinical and only two managerial skills. The 22 residents reported the contributions of preceptorship in the development of competences centered on clinical practice, focusing on technical procedures disjointed from clinical reasoning and the managerial dimension of the nurse’s work.

**Final Considerations:**

it is necessary to train preceptors and involve all social actors linked to residency programs to expand preceptorship potential.

## RESUMO

**Objetivos:**

analisar a experiência de enfermeiros residentes com as contribuições da preceptoria para o desenvolvimento das competências clínicas e gerenciais comuns previstas nos projetos pedagógicos.

**Métodos:**

pesquisa qualitativa exploratória desenvolvida em duas etapas: análise documental dos projetos pedagógicos e entrevista semiestruturada com residentes. Realizou-se análise de conteúdo fundamentada no referencial do processo de trabalho do enfermeiro e de competências.

**Resultados:**

os projetos pedagógicos dos três programas preveem o desenvolvimento de competências comuns majoritariamente clínicas e apenas duas competências gerenciais. Os 22 residentes relataram as contribuições da preceptoria no desenvolvimento de competências centradas na prática clínica, com foco em procedimentos técnicos separados do raciocínio clínico e da dimensão gerencial do trabalho do enfermeiro.

**Considerações Finais:**

faz-se necessária a formação dos preceptores e o envolvimento de todos os atores sociais vinculados aos programas de residência para ampliar o potencial da preceptoria.

## RESUMEN

**Objetivos:**

analizar experiencia de enfermeros residentes con las contribuciones de la preceptoría para el desarrollo de las competencias clínicas y gerenciales comunes previstas en los proyectos pedagógicos.

**Métodos:**

investigación cualitativa exploratoria desarrollada en dos etapas: análisis documental de los proyectos pedagógicos y entrevista semiestructurada con residentes. Realizado análisis de contenido fundamentado en el referencial del proceso de trabajo del enfermero y de competencias.

**Resultados:**

los proyectos pedagógicos de los tres programas prevén el desarrollo de competencias comunes mayormente clínicas y solo dos competencias gerenciales. Los 22 residentes relataron las contribuciones de la preceptoría en el desarrollo de competencias centradas en la prática clínica, con enfoque en procedimientos técnicos separados del raciocinio clínico y de la dimensión gerencial del trabajo del enfermero.

**Consideraciones Finales:**

es necesaria la formación de los preceptores y el involucramiento de todos los actores sociales vinculados a programas de residencia para ampliar el potencial de la preceptoría.

## INTRODUCTION

Residency programs in health in Brazil may be uni-professional or multi-professional, with a minimum duration of two years and full-time exclusive dedication, with 80% of the workload dedicated to in-service teaching (practice) and 20% to theoretical and theoretical-practical activities. Throughout the residency, the residents must develop skills provided in the pedagogical project (PP) of the program, obtaining the title of specialists in the area of expertise at the end of the course^(1)^. In nursing, the residency becomes a relevant stage in the training of professionals, since the National Curriculum Guidelines (NCGs) provide for a generalist training in undergraduate studies^(2)^.

Studies on the work process in nursing show, in Brazil and in other countries, two dimensions of action: healthcare and management^(3-4)^. However, the analysis of the contradictions of nurses’ practices shows the presence of denial of the managerial sphere, which tends to hide the uniqueness of their work, which resides in the inseparability of the dual dimension of care and management^(4)^.

Based on the framework, the study set out to investigate the clinical and managerial competences present in the PPs and the experience of resident nurses (called, in short, “residents”) regarding their development, in particular, with support from preceptors. “Clinical competences” are investigated instead of “healthcare”, as called in nursing work studies, because, in the country and in the global scenario, the literature highlights the expansion of nurses’ clinical practice both in specialized care^(5)^ and in primary care^(6)^, as well as the introduction of Advanced Practice Nursing^(6)^. Since the introduction of the Family Health Strategy in Brazil in the mid-1990s, nurses have not limited their work to the managerial dimension and started to perform clinical care activities in nursing consultations, home visits and others^(6)^. The denomination “clinical competences” was also adopted because the PPs studied refer to them.

In this study, Zarifian’s conception of competence was chosen, which he describes as taking the initiative, assuming responsibility, mobilizing practical intelligence and networks of actors around the situations, events and problems that the professional recognizes and seeks to face in everyday work^(7)^.

Clinical skills are related to the care dimension of the nurse’s work process, involve the ability to provide comprehensive and humanized care, technical procedures and other actions, as well as knowledge about pathologies and their implications for the patient’s conditions and others^(8)^. Managerial skills are those related to the organization and control of the work process, aiming to create and maintain adequate conditions for carrying out care based on the instruments: communication, supervision, decision-making, teamwork, continuing education, human resource management, material and physical, among others^(9)^.

The development of clinical and managerial skills can be facilitated through the preceptor’s work. This is the professional who carries out “direct supervision of the practical activities of residents in the health services where the program is developed [...], with a minimum specialist training”, and must be “linked to the training or executing institution” and belong to the “same professional area of the resident under their supervision”^(1)^. The main attributions of the preceptor include: providing guidance in the “performance of practical activities experienced in the daily routine of health care and management” and “identifying difficulties related to the acquisition of competences foreseen in the PP of the program [...]”^(1)^.

The preceptor is appointed as a facilitator of the residents’ training process, by providing opportunities for theoretical-practical reflection and exchange of experiences between professionals from different areas^(10)^. On the other hand, research on preceptorship in multi-professional and uni-professional residency programs point to some weaknesses, such as lack of pedagogical training of preceptors for supervision^(11)^, insufficient number of preceptors specialized in the area to monitor residents in the fields of practice^(12)^ and lack of articulation between theory and practice^(13)^.

It is understood that analyzing the preceptorship’s contributions to the development of clinical and managerial skills provided for in the PPs, from the perspective of residents, can contribute to the nursing area and other health areas, by supporting reflections and interventions that help to improve the training of resident professionals and the performance of preceptors in the context of hospital practice.

## OBJECTIVES

To analyze the residents’ experience with the preceptorship’s contributions to the development of common clinical and managerial skills foreseen in the three PPs studied, considering the context of hospital practice.

## METHODS

### Ethical aspects

This study complies with Resolutions No. 466/2012^(14)^ and No. 510/2016^(15)^ of the National Health Council and was approved by the Research Ethics Committee (REC) of the institution where it was developed. All participants received information about the study and signed the Free and Informed Consent Form. When presenting the results, the name of the participants was replaced by the letter I (Interviewee) followed by a numeral (1, 2, 3…) corresponding to the order in which the interviews were carried out.

### Study type

Exploratory qualitative study developed in two stages: document analysis and semi-structured interview. We opted for the development of field research and analysis in two stages (document analysis of the PPs and interview), in order to print greater consistency to the results based on the comparison between both results, the analysis of the residency programs and the reports about the residents’ experience. The nurses’ work process^(4)^ and Zarifian’s (2001)^(7)^ conception of competences were used as a theoretical reference. The competences were differentiated into two groups according to the dimensions of the nurse’s work: clinical and managerial. *The Consolidated criteria for reporting qualitative research* (COREQ) was used as a validation guide in the preparation of the study^(16)^.

### Methodological procedures

#### Scenario and study participants

The research analyzed three uni-professional residency programs in nursing at a public university located in the Southeast Region, namely: Obstetrics (OBS), Child and Adolescent Health (CAH) and Adult and Elderly Health (AEH). The activities of these programs began in 2013; and, annually, six places are offered per program. First-year residents are called R1; and those of the second, of R2. Active residents during the study period were invited via email to participate in the survey.

#### Data collection, organization, and analysis

In the document analysis, the PPs of the three uni-professional residency programs in nursing, the schedules of activities to be developed in each field of practice and the procedures validation and evaluation forms (self-assessment and hetero-assessment) were analyzed. The PPs were made available, via e-mail, by the secretariat of residency programs. The other documents were obtained by requesting residents of the three programs.

Bardin’s^(17)^ content analysis was used to identify the clinical and managerial competencies provided for in the PPs of the three programs, based on deductive analysis. A floating reading of the PP of each Program was carried out, followed by an exhaustive reading, coding, and classification of the clinical and managerial competences. A cross-sectional analysis of the three PPs was carried out, preparing a synthesis of clinical and managerial skills common to them. The analysis process was carried out by the first author, accompanied and validated by the second author, and recorded in an Excel spreadsheet.

A script was used in the semi-structured interviews^(18)^ with the residents of the three programs, consisting of questions related to the characterization of the participants and questions about the learning experience with the preceptorship, such as: significant situations for learning, teaching-learning strategies used, facilitators, obstacles and strategies for the development of preceptorship.

The interviews took place between January and April 2021, with an average duration of 25 minutes, a minimum of 13 and a maximum of 46 minutes. They were scheduled according to the availability of the 22 residents who agreed to participate in the research, in the online format^(14)^ or in person^(8)^. Online interviews were conducted via Google Meet, with the link sent via email; the face-to-face sessions took place at a date, time and place previously agreed with the participant (residents’ room at the hospital, participant’s home, outside the hospital - garden). In both situations, the recording was performed and later transcribed in full. All interviews were conducted by the first author, a resident of one of the programs studied, and third parties were not present. After the first two interviews, the wording of one of the questions in the script was changed to make it more objective and clearer. Observations and impressions obtained during this stage were recorded in the researcher’s field diary.

Bardin’s^(17)^ content analysis of the 22 transcripts of the interviews was carried out based on the same procedures described above: floating and exhaustive reading of the transcripts, followed by the codification and construction of the categories. These were prepared based on the comparison between the selected and coded excerpts of each interview and after the set of interviews, having as reference the conceptual theoretical framework adopted, the results of the document analysis and the search for convergences and divergences of the residents’ reports. The analysis was stored in an Excel spreadsheet and validated by the second author.

## RESULTS

### Analysis of pedagogical projects: clinical and managerial skills provided for in the pedagogical projects

Eight clinical competences and two managerial competences common to the three PPs were identified (Chart 1).

**Chart 1:** Summary of competences common to the three programs, based on documental analysis of PPs and procedure validation sheets, São Paulo, São Paulo, Brazil, 2022

Clinical skills
Humanized care centered on the patient and family; Evidence-based practice; Responsibility; Clinical reasoning; Ability to diagnose and propose nursing interventions; Domain of procedural techniques; Practice based on the principles of bioethics, ethics and legislation in nursing; Process of critical reflection and decision-making about care and care management.
**Managerial skills**
Development of skills related to multidisciplinary teamwork; Theoretical and practical subsidies referring to the organization and dynamics of work in the Health Care Network of the specific area of each program.

Common clinical skills predominate across the three PPs. It was noted that the PP of the OBS area describes clinical competences to deal with care that involve the woman’s reproductive health process in prenatal care, labor and birth, puerperium, newborn, and family, but does not refer to critical situations such as unwanted/planned pregnancy, violence, or abuse. The PPs in the areas of CAH and AEH list competencies related to comprehensive care, considering the specificities of children and adolescents, as well as adults and the elderly.

### Analysis of the interviews

#### Profile of participants

Of the 31 active residents invited to participate in the interviews, 25 responded to the invitation and 22 participated. As for the losses, six did not respond to the invitation, and three were unable to participate due to scheduling incompatibility.

There were 12 R1 and 11 R2, eight of which belonged to the OBS program; seven, CAH 16), without children (n = 20), who had attended a nursing degree at a public education institution (n = 19).

#### Residents’ experience with preceptorship and its contribution to the development of common clinical and managerial skills provided for in the pedagogical projects

The analysis of the interviews resulted in the construction of two categories: The residents’ experience with preceptorship; and Development of clinical skills with a procedural focus disjointed from clinical reasoning and the managerial dimension.

#### Residents’ experience with preceptorship

Most residents report that there was no formal introduction or mention of the names of the nurses who would be preceptors during their stay in the hospital units. Only three participants mentioned that, in some sectors, the nurse presented herself as a preceptor.

*“Oh, I’m a preceptor, this person is a preceptor”, that doesn’t happen.* […] *Yes* […] *if I had not read the portfolio* [Orientation Manual]*, I would not know who my preceptors are in the unit, and there is no formal presentation.* (I01)

In the residents’ view, the way preceptorship is carried out depends on the sector and the profile of the professionals who are on duty. Some reported having received more preceptorship from nursing technicians and nurses who were not formally preceptors, as well as not having perceived direct monitoring and supervision of activities developed in their teaching-learning process.

Most participants brought the experience that the preceptor’s role was more present and evident when the end of the period of passage in the units approached, a period in which it was necessary to complete the filling in of the schedule of activities carried out in the sector as well as the forms of validation of procedures and carry out the final evaluation. Some residents indicated the need for partial feedback in order to identify points to be improved before completing the internship in the sector; however, when requesting this, they did not receive a response most of the time.

*I went to the preceptor and asked if there was anything he wanted to tell me. But nobody ever called me. And the answer they gave was like this, that if they had something to say, surely, they would have called me before, that’s why they never would have called me.* […] *because, when they tell you that, you understand that everything is fine, and you know that you have points that need improvement.* […] *Then the evaluations happen, generally, at the very end of passing through the practicum fields.* […] *Then, in the end, something always comes up.* (I11)

On the other hand, learning experiences with preceptors were reported through welcoming attitudes and perception of interest in teaching, expressed by professionals when they reserve moments for discussion of cases and nursing conducts during the shift.

*People who want to be, who like to teach, who like to welcome us, are great preceptors. And anyone who doesn’t like it isn’t a preceptor. And as I said, the technicians, they give a show of preceptorship. Most of them. I prefer to ask, sometimes, a technician than a nurse. Things a nurse should guide me.* (I08)

[…] *He* [nurse] *would stop everything he was doing, so* […] *he could* […] *manage his time, making his time reserved for us to discuss, let’s say, blood gas testing, which has already happened. Discuss, he shows us how a ventilator works.* (I03)

Residents referred to the preceptors’ difficult working conditions, especially due to the shortage of human resources and the workload of nursing professionals. Some reported the experience of frequently having to take on direct assistance to cover staff shortages in the unit. They also mentioned the impact of the covid-19 pandemic on their experience as residents, especially R1, who only experienced the pandemic scenario. Residents brought up the fragility of teaching-service integration as a barrier to the development of preceptorship.

[…] *it is a hospital that is currently experiencing a nursing human resources crisis, and we end up being in care more often not because of a learning process, but because they need people to work and there are none. So, our learning process in relation to the role of the nurse itself, which is a more managerial process, for me it has been very, very compromised.* […] *And I feel that, when the person shows me the process, he is more concerned that I learn to do it in his place or to do it to help him, because he can’t handle it, than actually making me develop a logical reasoning and manage to learn the applicability of that, you know?!* (I21)

#### Development of clinical skills with a procedural focus disjointed from clinical reasoning and the managerial dimension

Residents evidenced the predominance of experiences related to the execution of technical procedures. They highlighted that, during the first execution of a certain technique in the unit, they had the support of the preceptorship, however, they mentioned that, usually, the supervision of technical procedures was not carried out in a way that allowed the development of clinical reasoning related to its execution.

[…] *it’s not just staying with me to pass a bladder catheter. It helps me in the interpretation of exams that are almost non-existent* […]*. I don’t just want practice; I don’t just want them to teach how to practice. I also want clinical reasoning. I think clinical reasoning is what is most lacking. So help me in that context.* (I18)

Fragility was also mentioned in the learning experiences about the nursing process and the articulation of the care and managerial skills of the nurse’s work.

The findings of the interviews showed divergences with the analysis of the three PPs, in particular regarding the lack of mention by the interviewees of teamwork and the decision-making process - both management tools, nursing care and management of care.

*So, it is during care procedures that I will be able to practice a dressing, that I will learn about a dressing, that I will learn about care, learn how to bathe the bed.* […] *After I was confident about it, after I had already practiced, I don’t know, one, two, three weeks, then I would have a moment to learn the work of the nurse, of the nurse. That it would be a job more of the nursing process, a more managerial job, a job of looking at your team and being able to nominate and really guide the team that I have.* […] *Then that person, along with the whole team, was responsible for showing the role of nurses themselves, this managerial role* […] *of being able to apply the nursing process not only with patients, but being responsible and having a role of leadership with the team. And I think this is the biggest gap in the residency.* (I21)

Although most residents did not report close and frequent preceptorship follow-up, they experienced situations that positively impacted their learning process and professional development. They cited moments in which the nurse and the nursing technician called them to participate in some urgent/emergency care or to perform some new procedure and discussed nursing conduct. The experience narrated by residents shows that direct supervision helped them to overcome difficulties and fears related to performing procedures and to act professionally as nurses. Some indicated the encouragement to study and the search for improvement as learning components.

*A complication appears, I may be doing something there,* […] *this nurse comes to me and says: “*[So-and-so]*, this complication arrived, I think it’s worth going there to see it for you to learn, it’s a new thing.” Then, she gives me the freedom to stop everything I’m doing, and I go there to see what’s going on. And then* […] *if there’s a procedure, for example, that I’ve never done, she teaches me! If there is any pathology that I am in doubt about, I know I can go and discuss it with her, and she can solve my doubts as well.* (I03)

Participants made suggestions to improve preceptorship contributions in the development of clinical and managerial skills of residents: building greater alignment of expectations, objectives and actions between the training institution, the hospital’s nursing management, nursing professionals and preceptors; training for preceptors, including clarifying the roles of the professionals involved; promote discussion of cases and nursing behaviors to assist in the development of clinical reasoning, evidence-based practice and the decision-making process; evaluation of preceptors by residents with feedback; stimulus for the inclusion of social skills, with an emphasis on communication present in the teaching-learning process carried out by preceptors.

## DISCUSSION

In the analysis of the competences foreseen in the PPs of each program and in the procedures validation forms, it was noticed during the residency period the predominance of the quest for the development and improvement of clinical competences with a procedural focus in detriment of the managerial ones, as presented in the results. This was also found in the results of the analysis of the interviews, in which we sought to know both the residents’ experience with preceptorship and its contribution to the development of the competences provided for in the PPs. Although the PPs foresee the development of the resident’s clinical reasoning in the face of the medical diagnosis and the survey of the patients’ nursing care needs, articulated with managerial skills, this was not observed in the present study.

According to the residents’ experience, nursing technicians and nurses who have welcoming attitudes and show an interest in teaching contribute significantly to their learning process. This result corroborates another study^(19)^, which showed that it is essential for the preceptor to welcome the residents and actively listen to their fears, anxieties and difficulties, as this helps them to feel cared for and acquire security in carrying out activities related to hospital nursing practice.

Although nursing technicians are not responsible for developing preceptorship, the results show that they also act as facilitators during this process, so it is desirable that they are recognized and encouraged to contribute to the training of residents. The participation of technicians in preceptorship is based on: their skills and dexterity in performing technical procedures that many nurses do not have; in its performance in a teaching hospital, which has specific characteristics of supporting education; and the opportunity that the interaction between residents and nursing technicians provides for the development of skills related to communication from the perspective of nursing teamwork^(20)^.

Several studies highlights the role of the preceptor nurse as a facilitator, reference guide and knowledge mediator in the training of nursing residents^(5,12,21)^. However, the following are considered as barriers to the effective practice of preceptorship: the lack of training of these professionals; the lack of knowledge about their attributions, the legislation and the governing PPs of the residency programs; work overload; and demotivation. This may be related to the addition of preceptorship to nursing professionals’ workday without acknowledging preceptorship activities as part of their work or receiving some incentive, such as extra remuneration or reduction of workload in other planned activities^(12,21)^.

Considering the global and national trend of education and interprofessional work, attention is drawn to the invisibility of preceptorship in nursing in view of the social recognition of preceptorship in medical residency, due to the fact that medical preceptors receive gratification since mid-2012^(22)^. Only in 2021, after the creation of the National Plan for Strengthening Residencies in Health within the scope of the Unified Health System (SUS), did discussions begin on granting a financial incentive to teachers, tutors and preceptors linked to uni-professional and multi-professional residency programs in health, including medical residency^(23)^. In this sense, the role of *SUS Escola* is highlighted, recognized as a space for learning and qualified training of health professionals.

Although the hospital practice settings under study had a staff of nurses composed mostly of specialists and some masters and doctors, residents reported weaknesses in the exercise of preceptorship. A study^(19)^ pointed out that nurses with a degree cannot be considered specialists in preceptorship if they do not develop skills to mobilize knowledge, skills and attitudes that recognize the educational needs of residents and support them in the learning process. It is up to the training institutions and executors of the residency programs, together with the Ministry of Health, to promote training actions for tutors and preceptors in order to prepare them to perform their functions^(1,23)^.

As for the contributions of preceptorship in the development of clinical competences related to healthcare, the study showed that the residents’ experience regarding preceptorship is, above all, support in the execution of technical procedures, often disjointed from the clinical reasoning that would allow understanding the relationships between the technique and conditions of the pathology, the living conditions of the patient and family, as well as, through them, defining nursing care.

A study^(5)^ carried out with nursing graduates pointed out that learning was centered on the development of clinical skills, mainly on the execution of procedural techniques and theoretical knowledge focused on managerial skills, whose application took place after training and insertion in the market of work. Another research^(19)^ highlights the role of the preceptor as an educator both when carrying out procedures with the objective of demonstrating them to residents and when helping them to articulate the techniques to the scientific knowledge that underlies them, which allows the development of knowledge and skills to act competent of the professional.

The three PPs presented only two managerial skills related to teamwork and the acquisition of theoretical and practical subsidies related to the organization and dynamics of work in the Health Care Network. However, the residents’ experience with preceptorship differs from the PPs, given the absence of teamwork and the decision-making process, the latter related in the three PPs to both care and care management. This result reinforces the emphasis on clinical competences and highlights the fragility of the articulation between clinical and managerial training in hospital preceptorship. The residents’ reports show their expectation that the residency would allow them to put themselves in the nurse’s shoes, developing integrated clinical and managerial skills. However, the narrated experience shows contradictions and tensions present in the nurses’ work process, both in specialized care and in Primary Health Care, referring to the fragmentation between clinical care and management in nursing work^(3-4)^.

Studies of nursing from the point of view of the work process, taking Collective Health and Social Sciences as a theoretical framework, consider nursing as a social and technical practice that is constituted in the dialectical historical process. This gives rise to health practices in modernity, especially since the hospital reform process, at the turn of the 18th to the 19th century, when they moved from the condition of a locus of asylum and death to a space of treatment and cure^(24)^.

The practice of nursing in modernity is formed with the internal division process of the area, therefore, configuring a heterogeneous practice, with two agents: the nurse and a professional assistant to the nurse. This model is reproduced until today with several agents in all countries^(25)^, remembering that the technical division is always subsumed to the social division of labor^(20,26)^.

There are studies indicating the existence of two dimensions in the nurses’ work process: assistance, which encompasses the clinical scope of nursing care; and managerial^(3-4,9)^. A recent study based on an international literature review^(4,27)^ advances in this direction and shows that the nurses’ work is inseparably care and management and that the “denial of the dual nature of work by the nurses via a profound process of alienation culminates in not recognition of self at work and its uniqueness”^(27)^. In addition, tension is observed in the supposed centrality of a single idealized nursing care between the different nursing agents who are under the coordination of the nurse.

Differences between the competences provided for in the PPs and the experiences reported by residents are related, in part, to the working conditions of nursing professionals in the study’s practice scenario. The shortage of human resources and work overload affect preceptorship, leading to the misuse of residents to take on the direct care of patients. Another research^(12)^ warns of possible conflicts of interest in health and teaching institutions, which are intended to function as a practice setting for the development of the competences provided for in the PPs, but can make use of residents as resources to face the shortage of nursing professionals.

On the one hand, assuming direct patient care enables residents to improve procedural technical skills. However, on the other hand, without the clinical reasoning associated with its execution, learning about the systematization of nursing care is limited, which includes the history, the survey of problems, the definition of diagnoses and nursing interventions; and all of this characterizes comprehensive care supported by critical reflection and evidence available in the scientific literature.

The results show the fragility of teaching-service integration, identified as a barrier to the development of preceptorship and corroborated by a study^(28)^ carried out with preceptor nurses of undergraduate Nursing courses, by showing that one of the challenges for teaching-service integration is the facing the duality between theory and practice.

The suggestions for improving preceptorship brought by the participants of this study were related to: the discussion of cases and nursing conduct, as it contributes to the development of the resident’s clinical reasoning and critical reflection; the training of preceptors for the practice of preceptorship that encourages the development of clinical and managerial skills; and investment in teaching-service-community integration. The implementation of the suggestions requires the participation of all social actors linked to the residency programs, including federal instances. In particular, the need to involve the coordinators and tutors of the educational institutions responsible for the programs and the managers and preceptors of the residents’ practice scenarios is highlighted, so that, together, they seek to build more potent opportunities for learning and also for acknowledging and strengthening preceptorship.

### Study limitations

The document analysis stage was limited due to the similarity between the three PPs, so that it was not possible to better explore the specific competences of each area of nursing knowledge and practice (OBS, CAH, AEH). Another limitation concerns the study design, which only included residents and not the preceptors of the three programs. The inclusion of preceptos, if it had occurred, would allow a more comprehensive understanding of the study problem through the triangulation of the results.

### Contributions to the area of nursing, health, or public policy

The study results contribute to possible adaptations in the nursing residency programs studied and also in other programs in the country. This is because there was consistency and coherence in the comparison between the reports of the experiences of the 22 participating residents, as well as these with results from other studies on the topic of preceptorship in residencies, in which their strengths and weaknesses are pointed out.

## FINAL CONSIDERATIONS

The PPs of the three studied programs indicate the predominant development of clinical competences, compared to managerial competences. The residents’ experience with preceptorship evidenced its focus on procedural clinical skills, with fragile articulation with clinical reasoning and the managerial dimension of nursing practice in the hospital setting. Some reports also showed learning experiences promoted by formal and informal preceptors, including nursing technicians, which enabled the development of broader clinical skills and confidence for practicing as a nurse. The limitations experienced with preceptorship were related, among other reasons, to the need for training for preceptorship, the shortage of nursing professionals and their overload in the context of the hospital practice studied.

## Data Availability

https://doi.org/10.48331/scielodata.IAREB9
